# Supplementary material for: GPI-80 Augments NF-κB Activation in Tumor Cells
Source: Int J Mol Sci. 2021 Nov 6;22(21):12027. doi: 10.3390/ijms222112027 (PMC8584666; doi:10.3390/ijms222112027)
Supplement: Supplementary file 1 [file ijms-22-12027-s001.zip › ijms-1420610 Supplementary Materials.pdf]

Supplemental "Materials and Methods"

Cells and cell culture

Human bladder cancer cell lines (HT1376, RT-4, SCaBER, and T-24), human prostate cancer cell lines (PC3, DU145, and LNCaP) and human kidney cancer cell lines (A-704 and Caki-1) were purchased from ATCC (Manassas, VA, USA). HEK293T cells were gifted by Prof. Ishii (Department of Immunology, Tohoku University). These cells were cultured in RPMI 1640 (Thermo Fisher Scientific, Waltham, MA, USA) and supplemented with 10% (v/v) heat-inactivated fetal calf serum (Biowest, Nuaillé, France), 5 mM L-glutamine, 50 U/mL penicillin G potassium, and 50 μg/mL streptomycin sulfate. These cell lines were cultured at 37°C with 5% CO2 at high humidity.

Reverse transcription polymerase chain reaction (RT-PCR)

Total RNA from these cells was purified using TRIzol RNA Isolation Reagent (Thermo Fisher Scientific) according to the manufacturer's instructions. Complementary DNA (cDNA) was synthesized from total RNA using the ReverTra Ace qPCR RT Master Mix with gDNA Remover kit (Toyobo, Osaka, Japan), and the cDNA was amplified using PCR as follows: denaturation at 98°C for 10 s, annealing at 55°C for 30 s, extension at 72°C for 40 s, and 35 cycles of heating at 98°C for 1 min. The primer sequences of GPI-80 were 5′-GAC TCC ACA TGT CCT CCT AAT G-3′ and 5′-GTT CTG TGA AGT TGA ACC CAT CCC TGG-3′. The PCR conditions and the primer sequences of human β-actin have been described previously [39].

Measurement of GPI-80 expression using flow cytometry

The expression of GPI-80 was measured using flow cytometry as described in our previous reports [40]. Briefly, the cells were removed using a non-enzymatic cell dissociation solution (Biological Industries, Beit-Haemek, Israel) and cell scraper (Genesee Scientific, San Diego, CA, USA). The cells were then incubated with Phycoerythrin (PE)-conjugated anti-GPI-80 mAb (1 μg/mL; clone 3H9; MBL, Nagoya Japan) or PE-conjugated IgG1 isotype-matched control Ab (clone MOCP-21; BD Biosciences, San Jose, CA, USA) for 30 min at 4°C and washed with phosphate-buffered saline (PBS) containing 3% (v/v) fetal calf serum (FCS) and 0.1% (w/v) NaN_3. The cells were fixed with 1% formaldehyde in PBS and stored at 4°C. The samples were measured using the FACSCanto II cytometer (BD Biosciences). Data were analyzed by FlowJo software, version 7.6.5 (BD Biosciences).

Western blotting

The cells were lysed using UltraRIPA buffer (BioDynamics Laboratory, Tokyo, Japan) with gentle sonication on ice and centrifuged at 10,000 × g for 5 min at 4°C. The supernatants were collected, and the protein concentration was measured using the bicinchoninic acid (BCA) assay kit (Takara Bio, Kusatsu, Japan). These lysates were stored at −80°C until the assay was performed. Proteins (25 μg/lane) were separated on a 4–15% gradient gel using SDS-PAGE (Bio-Rad, Hercules, CA, USA) and subsequently transferred onto polyvinylidene difluoride (PVDF) membranes. The membranes were blocked with 5% skim milk and probed with anti-GPI-80 monoclonal antibody (mAb, 10 μg/mL; 3H9, MBL, Nagoya, Japan) and anti-β-actin mAb (2 μg/mL; sc3489, Santa Cruz Biotechnology, Dallas, TX, USA). The proteins were detected using horseradish peroxidase (HRP)-conjugated secondary anti-mouse antibody (1:1000; P0260, DAKO, Santa Clara, CA, USA) and ECL™ Prime western blotting detection reagent (GE Healthcare Life Sciences, Marlborough, MA, USA). The chemiluminescence reaction was detected using the EZ-capture II imaging system (ATTO, Tokyo, Japan).

Confocal microscopy

The cells were fixed with 1% formaldehyde in PBS for 30 min. After washing with PBS, the cells were incubated with 3% FCS for 30 min and then stained with APC-conjugated anti-CD29 mAb (5 μg/mL; clone TS2/16; BioLegend, San Diego, CA, USA), PE-conjugated anti-GPI-80 mAb (10 μg/mL; clone 3H9; MBL), Alexa 488-conjugated anti-FLAG mAb (5 μg/mL; clone FLA-1; MBL), and 4',6-diamidino-2-phenylindole (DAPI; 5 μg/mL, Merck, Darmstadt, Germany) overnight. For staining of phosphatidyl serine on the cell surface, cells were incubated with FITC-conjugated Annexin V (40 μg/mL, BioLegend) before cell fixation, instead of Alexa 488-conjugated anti-FLAG mAb. After the reaction, the cells were fixed and incubated with antibodies, as described above. For F-actin staining, the fixed cells were stained with unlabeled anti-GPI-80 mAb (10 μg/mL, 3H9) and then with rabbit F(ab')2 immunoglobulin FITC-conjugated anti-mouse IgG antibody (dilution, 1:20; F0313, DAKO). After the antibody reactions, the cells were permeabilized with 0.5% Triton X-100 for 5 min and incubated with Acti-Stain 670 phalloidin (100 nM; PHDN1; Cytoskeleton, Denver, CO, USA) and DAPI (5 μg/mL) for 30 min. The stained samples were washed with PBS and embedded in ProLong Diamond (Thermo Fisher Scientific). The embedded samples were observed by confocal microscopy (LSM 700; ZEISS, Oberkochen, Germany).

Sandwich enzyme-linked immunosorbent assay (ELISA)

Human plasma was isolated by centrifugation at 300 × g for 5 min at 4°C. The

conditioned medium was centrifuged at 2,400 × g for 1 min at 4°C, and then the supernatants were re-centrifuged at 10,000 × g for 30 min at 4°C. The plasma samples and the re-centrifuged supernatants were stored at -80°C until the assay was performed. The anti-GPI-80 mAb (3H9, 1 μg/well) was coated on ELISA plates (Sumitomo Bakelite, Tokyo, Japan) and kept for 16 h at 4°C. Non-specific binding was blocked by incubation of the samples with PBS containing 5% skim milk (Cell Signaling Technology, Danvers, MA, USA) for 30 min at 23-25°C. After removing the skim milk solution, 100 μL of each sample was added to each well and incubated for 1 h at 23-25°C. Each well was washed three times with 400 μL of washing buffer (PBS containing 0.05% Tween 20). The bound antigens were detected by incubating with anti-GPI-80 mAb (4D4, 1 μg/well; IgG2a subclass, prepared as described previously [41]) or anti-CD63 mAb (1 μg/well; 8A12, IgG2a subclass; COSMO BIO, Tokyo, Japan) for 1 h at 23-25°C, and then quantified by incubation with HRP-labeled goat anti-mouse IgG2a (dilution 1:3000; Ab97245; Abcam, Cambridge, UK) for 30 min at 23-25°C. After each antibody reaction, each well was washed four times with washing buffer, and the color was developed using TMB solution (100 μL/well, KPL, Milford, MA, USA). The color development was stopped with 1 M phosphoric acid (100 μL/well), and the absorbance was measured at 450 nm using a microplate reader (Sunrise Remote, Tecan, Männedorf, Switzerland).

Induction of GPI-80 expression by lentiviral vector

The expression vector, pLV-SIN/puro was purchased form Takara Bio. The FLAG-tagged GPI-80 construct was generated by overlap extension PCR using cDNA from differentiated HL60 cells with 1.25% dimethyl sulfoxide (DMSO) [42, 43]. The primer sequences are listed in Supporting Table S1 (as below). The top and bottom fragments were mixed and used as template, and overlap PCR was performed using GPI80/1T/*Xho* I and GPI80/1574B/*Not* I primers. The cDNA construct was designed by inserting the FLAG amino acid sequence (8 aa in length) at the signal peptide cleavage position (at 23rd aa) in GPI-80 cDNA, and the FLAG-tagged cDNA was ligated with the pLV-SIN/puro vector at the *Xho* I-*Not* I site (pLV-SIN/puro-GPI-80). The ligated sequence was confirmed by DNA sequencing.

Lentivirus packaging was performed as follows: pLV-SIN/puro vector and packaging vectors (pMD2.G, pRSV-Rev, and pMDLg/pRRE; Addgene, Watertown, MA, USA) were transfected into HEK239T cells using Lipofectamine 2000 (Thermo Fisher Scientific). The cells (~5 × 10^5 cells) were infected with lentiviral particles (infectivity titer was 2 × 10^6) in the presence of polybrene (10 μg/mL) for 48 h. After infection, the cells were immediately used for subsequent experiments. To establish a stable GPI-80-expressing clone, the cells were diluted and cultured with puromycin (2 μg/mL; InvivoGen, San Diego, CA, USA). After

puromycin selection, single colonies were selected. GPI-80 expression was confirmed by flow cytometry as described above.

Deletion of GPI-80 by gene editing using CRISPR-Cas9

The targeting sequence of GPI-80/VNN2 was identified using the website, "CRISPRdirect" (https://crispr.dbcls.jp/). The DNA oligomer containing the targeting sequence for ligation into the lentiCRISPRv2 plasmid vector (Addgene) was designed according to the manufacturer's instructions. The oligo sequences that target exon 4, oligo-1, CAC CGC TTT CAA CAC CGC ATT TGG A and oligo-2, AAA CTC CAA ATG CGG TGT TGA AAG C, were purchased from Sigma-Aldrich (St. Louis, MO, USA). These DNA oligomer pairs were annealed and ligated into lentiCRISPRv2 vector according to the manufacturer's instructions. The vector was co-transfected with packaging vectors as described above. The cells (5×10^4 cells) were infected with the lentiviral particles (infectivity titer, 2×10^5) in the presence of polybrene (10 μg/mL) for 48 h, and the cells were expanded without cell cloning. The deletion of GPI-80 expression was confirmed by flow cytometry as described above.

FLAG/GPI-80/Fc fusion cDNA construct

GPI-80 cDNA was produced by reverse transcription-polymerase chain reaction (RT-PCR) using total RNA from human peripheral leukocytes with a primer pair of p-10T-Hind III and p+1563B-Not I. GPI-80 cDNA was fused with FLAG and signal peptide sequence (SP) of the human Vanin-1 (VNN1) by overlap extension PCR method using high fidelity DNA polymerase, pyrobest® (Takara Bio). In the first step, the SP of VNN1 cDNA fused with FLAG cDNA at the 3' end (SPVNN1/FLAG cDNA) was produced by extension PCR using the template of EST clone No. 85812 with a primer pair of VNN1/1T/*Hind* III and VNN1/77B/FLAG. While, GPI-80 cDNA fused with FLAG cDNA at the 5' end (FLAG/GPI-80 cDNA) were also produced by extension PCR using the template of GPI-80 cDNA with a primer pair of GPI80/78T/FLAG and p+1563B-Not I. In the second step, the overlap PCR was performed using mixture of SPVNN1/FLAG cDNA and GPI-80/FLAG cDNA (as a template) with a primer pair of VNN1/1T/*Hind* III and p+1563B-*Not* I. The GPI-80 cDNA fused with SP of VNN1 plus FLAG sequence (V/FLAG/GPI-80 cDNA) was digested with *Hind* III and *Not* I, and then subcloned into the *Hind* III/*Not* I site of the pcDNA3/Neo (Invitrogen, San Diego, CA, USA). The nucleotide sequence of the subcloned PCR product was confirmed by the method described previously [4]. The sequences of primers used in this procedure were presented in Supporting Table S2 (as below).

The Fc cDNA of mouse IgG2a in pBlueScript SK (-) was generously provided by Dr.

Yagita (Juntendo University, Tokyo, Japan). For ligation with Fc cDNA, *Bam* HI site was extended at +1456 bp of V/FLAG/GPI-80 cDNA by extension PCR using primer pair of pcD/T and Fc/Bam HI. The Fc cDNA was digested with *Bam* HI/*Not*I, and V/FLAG/GPI-80-*Bam* HI PCR product was treated with *Hind* III/*Bam*HI. After purification with agarose gel, these fragment cDNAs were mixed and subcloned into the *Hind* III/*Not* I site of the pcDNA/Zeo vector (Invitrogen). The V/FLAG/GPI-80/Fc cDNA subcloned into pcDNA3/Zeo vectors are referred as pcD/VF/GPI-80/Fc. These nucleotide sequences were also confirmed.

Transfection of pcD/VF/GPI-80/Fc into CHO cells and purification of sGPI-80/Fc fusion protein

CHO cell line was obtained from American Type Culture Collection (Rockville, MD, USA). These cells were maintained at 37°C in 5% CO2 in RPMI 1640 supplemented with 10% FCS, 50 U/ml penicillin G potassium (Banyu Pharmaceutical, Tokyo, Japan) and 50 μg/ml streptomycin sulfate (Meiji Seika, Tokyo, Japan). CHO cells (4×10^6) were incubated with 10 μg pcD/VF/GPI-80/Fc plasmid in 300 μl culture medium for 10 min on ice. The mixture was transferred into the 0.4 mm gap cuvette. Electroporation was performed at 240 volt and 1050 μF in a Gene Pulser unit (Bio-Rad Laboratories, Hercules, CA, USA) in the condition of the time constants between 30.3 to 46.2 msec. The electroporated cells were kept on ice for 15 min and recovered in pre-warmed (37°C) 5 ml RPMI 1640 containing 10% FCS for 48 h. The cells were transferred to 96-well culture plates and selected by the dilution plating technique using Zeosin (final concentration, 0.5 to 0.8 mg/ml) for the establishment of stable transfectant clone (GPI-80/Fc/CHO-#20). The production of soluble GPI-80 fused with FLAG and Fc (sGPI-80/Fc) fusion protein in cultured medium was checked using sandwich ELISA as described previously [7]. For purification of sGPI-80/Fc fusion protein, GPI-80/Fc/CHO-#20 was cultured in ASF104 serum free medium (Ajinomoto, Tokyo, Japan). The conditioned medium of GPI-80/Fc/CHO-#20 was bound to Protein A sepharose FF column, and then eluted as an immunoglobulin fraction with 0.05 M glycine-HCl (pH 3.5). The eluted fraction was applied to FLAG affinity column and eluted as a FLAG tagged protein according to the manufacture's protocols. The purified fusion protein (approximately 94 kD in reducing condition) was confirmed by Western blotting using anti-mouse IgG Ab or rabbit anti-GPI-80 poly Ab. The purity of the fusion protein was verified as single band with Coomassie Brilliant Blue staining.

Cell adhesion assay

Cell adhesion assay have been described elsewhere [4]. Briefly, the transfected PC3 cells were seeded in 96-well culture plates at 2.5×10^4 cells/well in the presence of anti-GPI-

80 mAb (3H9) and incubated for 6 h. After incubation, the non-adherent cells were removed by gentle vortex, and the plates were stained with crystal violet solution (0.05% crystal violet, 10% ethanol, and 5% neutralized formaldehyde) for 30 min. After staining, the plates were washed using tap water and allowed to dry. The stained cells were lysed using 0.1% SDS and measured at an optical density (OD) of 595 nm using a plate reader. The data are represented as the mean ± standard deviation from duplicate, and the figure is representative of results from more than four independent experiments. Percentage of cell adhesion was calculated as follows: percent (%) of cell adhesion = (OD595 of each 3H9 mAb dose)/(mean from OD595 of 0 μg mAb) × 100.

Migration assay

#22mock and #22ΔGPI-80 cells were suspended in FCS starved culture medium and seeded onto 6.5 mm culture insert (pore size 8.0 μm; Corning, 3422) at 2.5 ×10^4 cells/0.1 mL/well. In the bottom of the 24-well plate, 0.65 mL of the culture medium containing 10% FCS with or without anti-GPI-80 mAb (10 μg/mL, 3H9) was added, and incubated for 24 h. After incubation, the inserts were stained with crystal violet solution, and then, the inside of the inserts were scraped using cotton swab. The migrated cells on the outside of the inserts were counted using microscopy.

Pantetheinase assay

Pantetheinase assay was performed as previously described [44]. Pantothenate-7-amino-4-methylcoumarin (pantothenate-AMC), that was synthesized by Prof. Asao (Shinshu University's Molecule and Material Synthesis Platform, Japan), was dissolved in DMSO and then diluted in 100 mM potassium phosphate buffer (pH 7.5) containing 0.01% BSA, 0.5 mM DTT, 1% DMSO, and 0.0025% Brij-35. The cells were washed three times with PBS and lysed with ultra-RIPA buffer (BioDynamics Laboratory). The cell lysate was centrifuged at 10,000 × g for 5 min at 4°C and the supernatant was used for pantetheinase assay. The protein concentration was measured using the bicinchoninic acid (BCA) protein assay kit (Takara Bio). The conditioned medium was collected from confluent cells and was centrifuged at 800 × g for 5 min at 4°C. The samples (10 μL) were mixed with 20 μM pantothenate-AMC (final volume, 200 μL/well in a 96-well plate), and the change in fluorescence was measured over a 90 min period at 25°C using multi-label reader ARVO-X3 (PerkinElmer, Waltham, MA, USA). The progress of the reaction was followed every 10 min by detecting the fluorescence (excitation 355 nm, emission 460 nm).

Supporting Tables

Supporting Table S1. The sequence of primers for production of FLAG/GPI-80 cDNA for lentivirus vector

Primer name	Primer sequence
GPI80/1T/*Xho*I	ATTCCTCGAGAAGAAGAAAAATGATGATAAAG
	Xho I
GPI80/191B/FLAG	CTTGTCGTCATCGTCTTTGTAGTCAGTACCAACCTGCAGGGTTATTAG
	FLAG sequence
GPI80/78T/FLAG	GACTACAAAGACGATGACGACAAGCAGGACAGTTTTATAGCTGC
	FLAG sequence
GPI80/1574B/*Not*I	TAGGCGCGGCCGCTATAACATTACAATATTTTGC
	Not I

The site of restriction enzyme or FLAG sequence are indicated as the underline.

Supporting Table S2. The sequence of primers for production of VF/GPI-80/Fc cDNA

Primer name	Primer sequence
p-10T-*Hind* III	5'-aggttaagcttAACCTTGGCCA+1TGGTCACTTCCTCTTTTCC-3',
	Hind III
p+1563B-*Not* I	5'-taggcgcggccgCTATAACATTACAATATTTTGCAAAGC-3',
	Not I
VNN1/1T/*Hind* III	5'-aggttaagcttCATTGGACTTCAGCATGACTACTCAG-3'
	Hind III
VNN1/77B/FLAG	5'-cttgtcgtcatcgtctttgtagtcGCAGCTGGCTCTTGAGACATAG-3'
	FLAG sequence
GPI80/78T/FLAG	5'-gactacaaagacgatgacgacaagCAGGACAGTTTTATAGCTGC-3'
	FLAG sequence
Fc/*Bam* HI	5'-gcgggatcCCTCCCAAAGAGTGACACTG-3'
	Bam HI
pcD/T	5'-GTGGGAGGTCTATATAAGCAG-3'
pcD/B	5'-GCAAACAACAGATGGCTGGC-3'

Small characters of the primer sequences show the non-aneling sequence to each template.

The site of restriction enzyme and FLAG sequence are indicated by the underline and each name.

Supplemental Figures

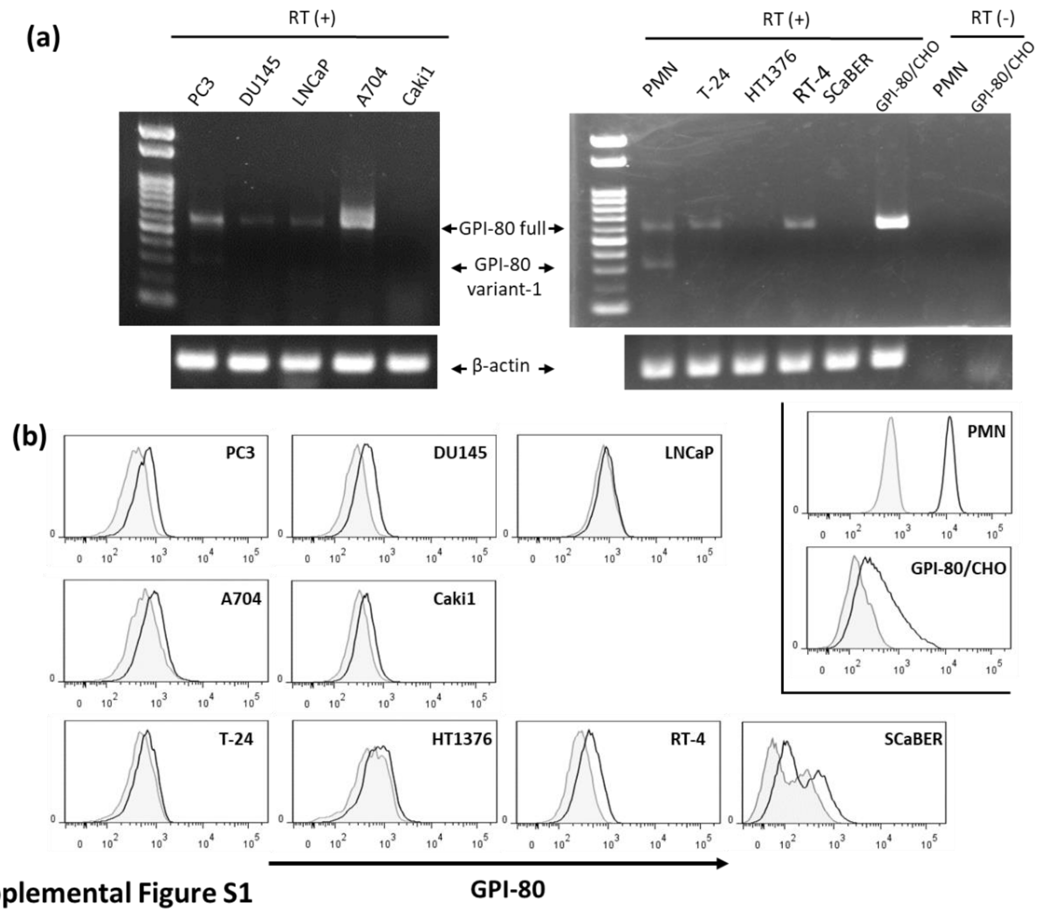

Supplemental Figure S1

Supplemental Figure S1. GPI-80 expression detected by RT-PCR and flow cytometry in urologic tumor cell lines. (**a**) Detection of GPI-80 mRNA expression. GPI-80 mRNA expression was detected by RT-PCR using total RNA from 3 prostate cancer cell lines (PC3, DU145, and LNCap), 2 kidney carcinoma cell lines (A-704 and Caki-1), and 4 urinary bladder cell lines (HT1376, RT-4, SCaBER, and T-24). For the control, polymorphonuclear cells and GPI-80 cDNA-transfected CHO cells were used. RT (+) or RT (-) indicate that reverse transcription reaction was performed in the presence or absence of reverse transcriptase, respectively. To check cDNA synthesis, PCR was also performed using β-actin primers. The GPI-80 primer pair was able to amplify both the full-length GPI-80 mRNA (GPI-80 full) and alternative spliced variant mRNA (GPI-80 variant-1) by PCR. The results are representative data from three independent experiments. (**b**) Detection of GPI-80 level on the cell surface by flow cytometry. The cells were stained with PE-conjugated anti-GPI-80 mAb (3H9) or isotype control Ab and analyzed by flow cytometry. The representative analysis includes data from more than three independent experiments.

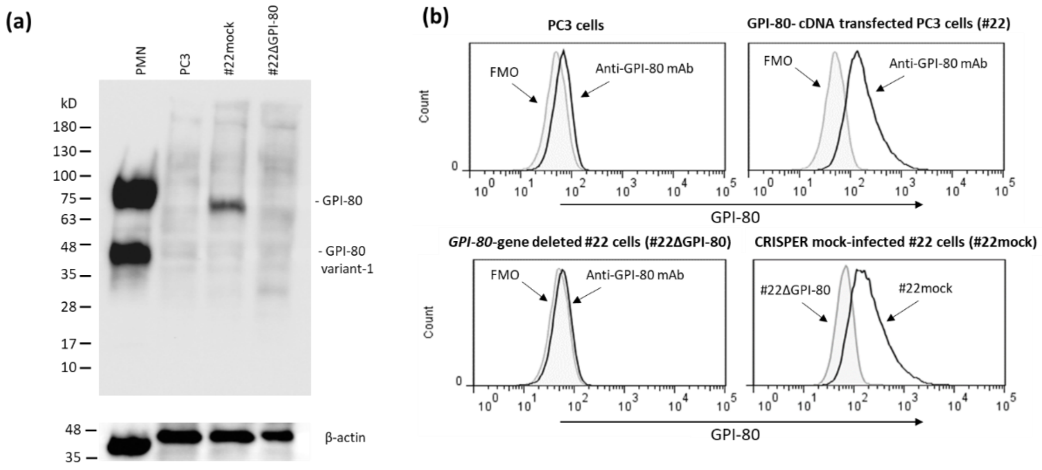

Supplemental Figure S2

Supplemental Figure S2. Levels of GPI-80 in PC3 cells and in PC3 transformants. (**a**) Detection of GPI-80 level by western blotting. Cell lysates (25 μg/lane) from PMN leukocytes, PC3 cells, #22mock cells (GPI-80-expressing PC3 transformant clone #22, which was transfected with CRISPR/Cas9 without the guide RNA sequence packaged with the lentivirus), and #22ΔGPI-80 cells (GPI-80 gene in #22 transformant was deleted by CRISPR/Cas9 lentivirus system) were subjected to electrophoresis, and western blotting was performed. The immunoblots were incubated with anti-GPI-80 mAb (3H9) or anti-β-actin mAb, and then the reactions were detected by HRP-conjugated anti-mouse antibody. The western blotting result is representative of results from two independent experiments. (**b**) Detection of GPI-80 level by flow cytometry. The fluorescence minus one (FMO) control was stained with PE-conjugated isotype-matched control mAb (IgG1). GPI-80 level was detected using anti-GPI-80 mAb (3H9). The upper left panel shows the result from parental PC3 cells; the upper right panel is the result from clone #22, which was infected with GPI-80 cDNA expression lentivirus construct; the lower left panel is from #22ΔGPI-80, and the lower right panel indicates the comparison of GPI-80 level between #22mock and #22ΔGPI-80 cells. These data are representative from more than three independent experiments.

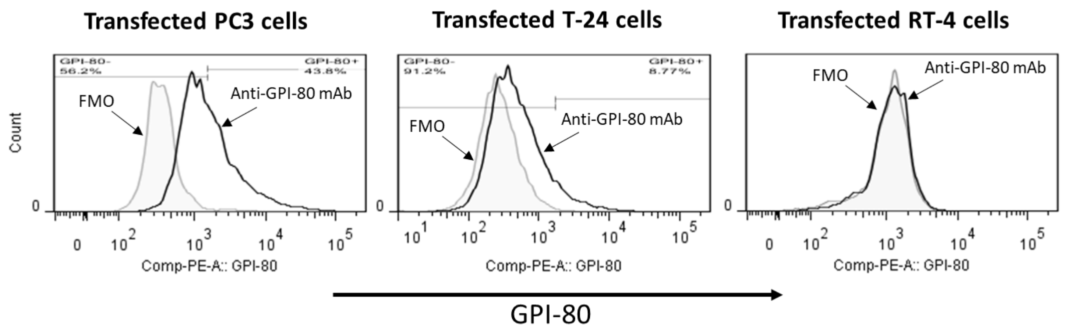

Supplemental Figure S3

Supplemental Figure S3. Detection of transient GPI-80 levels in PC3, T-24, and RT-4 cells. PC3 (left panel), T-24 (middle panel), and RT-4 (right panel) cells were infected with lentiviral vector with GPI-80 cDNA expression construct (GPI-80 cDNA ligated into pLVSIN-CMV Pur Vector packaged by 3 generation lentivirus packaging system). After selection using 10 μg/mL puromycin for 10 days, the cells were analyzed by flow cytometry. The histogram analysis of GPI-80 level is presented. The fluorescence minus one (FMO) control was incubated with PE-conjugated isotype-matched control mAb (IgG1), and the data are shown as gray histograms. The open histograms represent data of samples which were stained with anti-GPI-80 mAb (3H9).

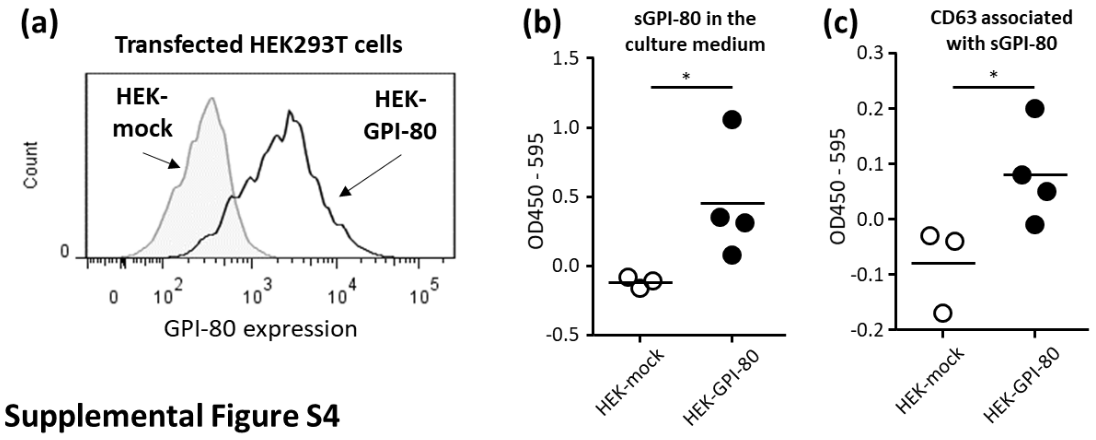

Supplemental Figure S4. The detection of soluble GPI-80 (sGPI-80) in the conditioned of GPI-80-transfected HEK293T cells. (a) GPI-80 expression in transfected HEK293T cells. HEK293T cells were transfected with lentiviral vector packaged with pLV-SIN/puro-mock vector (gray filled histogram, HEK-mock) or pLV-SIN/puro-GPI-80 vector (open histogram, HEK-GPI-80), and the infected cells were incubated for 2 days. After incubation, the transfected cells were stained with PE-conjugated anti-GPI-80 mAb (3H9) and analyzed by flow cytometry. HEK-mock (open circle) or HEK-GPI-80 (closed circle) were cultured for 3 days and the conditioned media were collected. (b) The media were used to perform sandwich ELISA to detect soluble GPI-80 using the paired anti-GPI-80 mAbs (3H9 and 4D4). (c) To detect the colocalization of GPI-80 with CD63, the media were analyzed using the paired anti-GPI-80 mAb (3H9) and anti-CD63 mAb (8A12). The data represent results from more than three independent experiments, and the statistical significance was calculated by two-tailed unpaired Student's *t*-test (*, $p < 0.05$).

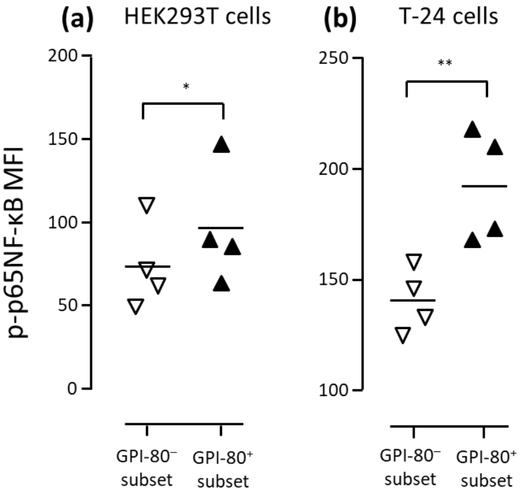

Supplemental Figure S5

Supplemental Figure S5. The relation between GPI-80-expressing cell subset and the activated NF-κB cell subset in HEK293T cells and T-24 cells. GPI-80^{-} cell subset and GPI-80^{+} cell subset were separated from (a) GPI-80-transfected HEK293T cells and (b) GPI-80-transfected T-24 cells as described in Figure 5. The p-p65 NF-κB MFI was analyzed in GPI-80^{-} cell subset (open in-versed triangle) and GPI-80^{+} cell subset (closed triangle). The data represent results from four independent experiments, and the statistical significance was calculated by two-tailed paired Student's *t*-test (*, $p < 0.05$; **, $p < 0.01$).

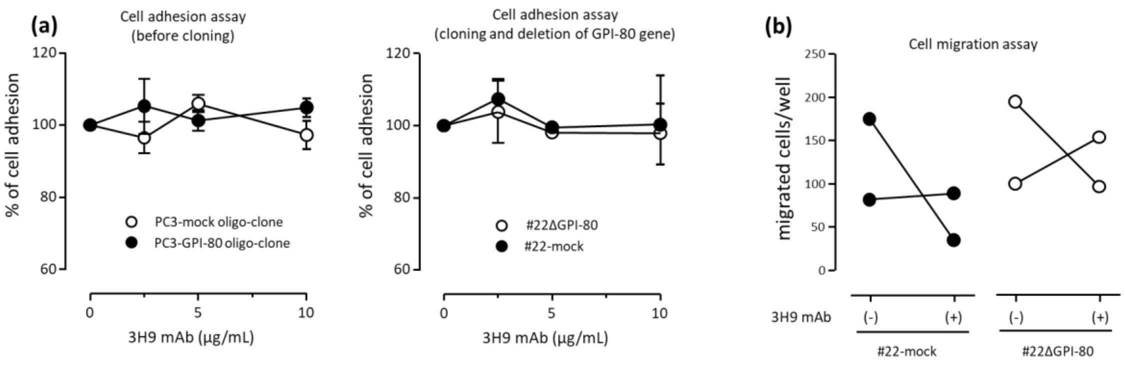

Supplemental Figure S6

Supplemental Figure S6. Cell adhesion and migration assay using transfected PC3 cells.

(**a**) Cell adhesion assay. The left figure represents data from oligo-clone; the right figure represents results from cloned #22mock and #22ΔGPI-80 cells. The cell adhesion assay data is representative of results from three independent experiments. (**b**) Migration assay. The migrated cells on the outside of the inserts were counted. The migration assay data is representative of results from two independent experiments.

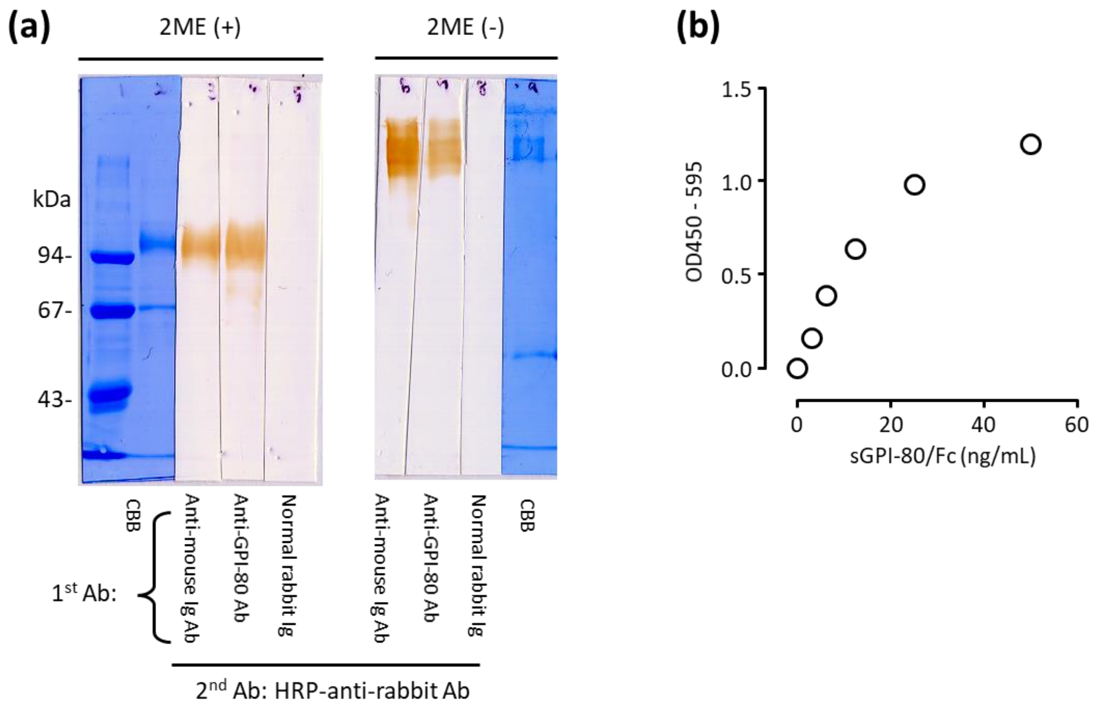

Supplemental Figure S7

Supplemental Figure S7. Generation of sGPI-80/Fc fusion protein. (**a**) Verification of sGPI-80/Fc by western blotting. The purified sGPI-80/Fc, which was prepared as described in Doc S1, was treated with (2ME +) or without 2-mercaptoethanol (2ME -), and then the samples (2.5 μg/lane) were electrophoresed using a 7.5% gel by SDS-PAGE and transferred onto a PVDF membrane and analyzed by western blotting. The membranes were stained with Coomassie Brilliant Blue R-250 (CBB) or immune blotted using rabbit anti-mouse immunoglobulin (1:500; Z0259, DAKO) or rabbit anti-GPI-80 antibody (15 μg/mL). For the negative control, normal rabbit immunoglobulin fraction (20 μg/mL; X903, DAKO) was used. The antibody reactions were detected using HRP-conjugated anti-rabbit antibody (1:1000; P0217, DAKO). Color development was performed using 3,3′-diaminobenzidine (DAB). (**b**) Detection of sGPI-80/Fc by sandwich ELISA. The purified sGPI-80/Fc was verified by sandwich ELISA using the paired anti-GPI-80 mAb (3H9) and HRP-labeled goat anti-mouse IgG2a (1:3000; Ab97245, Abcam).

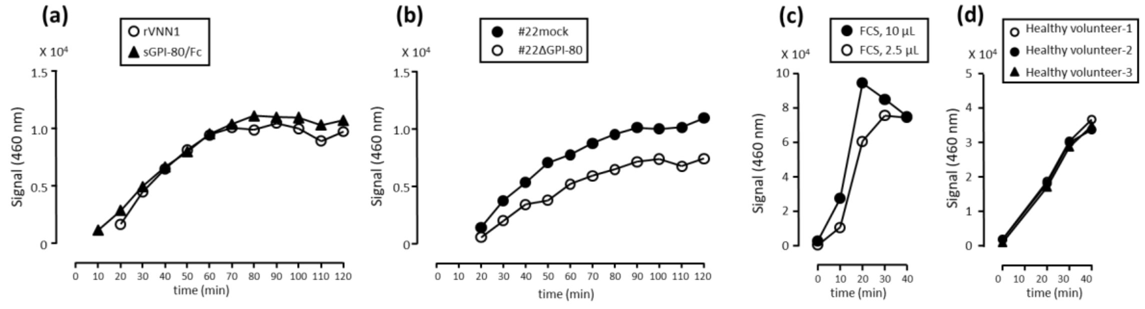

Supplemental Figure S8

Supplemental Figure S8. Pantetheinase assay of GPI-80. (**a**) Pantetheinase activity of purified recombinant proteins. Recombinant VNN1 (rVNN1, H0008876-P01) were purchased from Abnova (Taipei, Taiwan). Pantetheinase activity of rVNN1, (open circle, 0.125 μg/sample) and soluble GPI-80/Fc chimera (sGPI-80/Fc, closed triangle, 1 μg/sample) was measured. (**b**) Pantetheinase activity of cell lysate. The concentration of cell lysates from #22mock (closed circle) and #22ΔGPI-80 cells (open circle), was adjusted to 10 μg/sample and used for pantetheinase assay. The presented data are subtracted using the value of ultra-RIPA buffer (background of lysis buffer). (**c**) Pantetheinase activity in fetal calf serum (FCS). The pantetheinase activity in FCS (2.5 or 10 μL/sample), present in the cell culture medium, was measured. (**d**) Pantetheinase activity in human plasma. The pantetheinase activity in human plasma samples obtained from three healthy individuals (10 μL/sample) was measured. The data (mean of duplicate) are representative of results from more than three independent experiments.
